# Supplementary figures and images for: The Use of Electrochemical Voltammetric Techniques and High-Pressure Liquid Chromatography to Evaluate Conjugation Efficiency of Multiple Sclerosis Peptide-Carrier Conjugates
Source: Brain Sci. 2020 Aug 21;10(9):577. doi: 10.3390/brainsci10090577 (PMC7565688; doi:10.3390/brainsci10090577)

## Supplementary

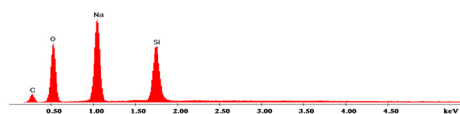

**Figure S1.** EDS elemental microanalysis of a Graphite/SiO<sub>2</sub> film electrode.

Supplement: Supplementary file 1 [file brainsci-10-00577-s001.pdf]
